# Accuracy of dynamic contrast-enhanced magnetic resonance imaging in the diagnosis of prostate cancer: systematic review and meta-analysis

**DOI:** 10.18632/oncotarget.20316

**Published:** 2017-08-17

**Authors:** Zhiqiang Chen, Yi Zheng, Guanghai Ji, Xinxin Liu, Peng Li, Lei Cai, Yulin Guo, Jian Yang

**Affiliations:** ^1^ Department of Radiology, The First Affiliated Hospital of Xi'an Jiaotong University, Xi'an 710061, Shaanxi, China; ^2^ Radiology Department of The General Hospital, Ningxia Medical University, Yinchuan 750004, Ningxia, China; ^3^ Department of Diagnostic Imaging, Honghui Hospital, Health Science Center of Xi'an Jiaotong University, Xi'an 750004, Shaanxi, China

**Keywords:** diagnostic imaging, diagnostic techniques, early detection of cancer, magnetic resonance imaging, male

## Abstract

The goals of this meta-analysis were to assess the effectiveness of dynamic contrast-enhanced magnetic resonance imaging (DCE-MRI) in patients with prostate carcinoma (PCa) and to explore the risk profiles with the highest benefit. Systematic electronic searched were conducted in database. We used patient-based and biopsy-based pooled weighted estimates of the sensitivity, specificity, and a summary receiver operating characteristic (SROC) curve for assessing the diagnostic performance of DCE. We performed direct and indirect comparisons of DCE and other methods of imaging. A total of 26 articles met the inclusion criteria for the analysis. DCE-MRI pooled sensitivity was 0.53 (95% CI 0.39 to 0.67), with a specificity of 0.88 (95% CI 0.83 to 0.92) on whole gland. The peripheral zone pooled sensitivity was 0.70 (95% CI 0.46 to 0.86), with a specificity of 0.88 (95% CI 0.76 to 0.94). Compared with T2-weighted imaging (T2WI), DCE was statistically superior to T2. In conclusion, DCE had relatively high specificity in detecting PCa but relatively low sensitivity as a complementary functional method. DCE-MRI might help clinicians exclude cases of normal tissue and serve as an adjunct to conventional imaging when seeking to identify tumor foci in patients with PCa.

## INTRODUCTION

Prostate cancer is the second most common form of cancer in males worldwide [1], and the growing elderly population has led to an increase in the number of estimated new prostate carcinoma (PCa) cases. PCa accounted for the highest number of estimated new cases of cancer and the second highest number due to cancer deaths of males in the United States in 2015, with totals of 220,800 and 27,540, respectively [2]. In the developed regions of East Asia (i.e., Korea, Japan, and the Taiwan and Shanghai regions of China), PCa is the fifth most common cancer as well as the most common genitourinary cancer, with approximately 9% (82,691) cancer cases diagnosed with PCa (8.2/100,000) [3]. By 2030, the global burden of PCa is expected to increase by 1.7 million new cases, and 499,000 deaths will occur because of population growth and aging worldwide [4].

PCa can initially develop slowly and remain limited to the prostate gland. However, some types develop quickly and become aggressive. Delays in the diagnosis of PCa reduce the likelihood of treating PCa in its early stages. Onwukamuche et al. showed that doctors only obtain a 36.4% sensitivity level with regard to the diagnosis of PCa [5], especially among patients who are in the early stages of this disease and exhibit no signs of illness. All available diagnostic tools for early detection, such as digital rectal examination (DRE), serum prostate-specific antigen (PSA; a nonspecific blood test) and transrectal ultrasound (TRUS)-guided biopsy, have limitations due to their nonspecific characteristics, their invasive nature, or both [6]. In 2014, Cambridge University established that as many as half of all patients who participated in their study were at risk of PCa misdiagnosis because of inaccurate examination techniques [7]. Therefore, an effective method for the early detection of PCa with high sensitivity and specificity, and noninvasiveness is urgently needed in the clinic.

Magnetic resonance imaging (MRI) shows much better spatial resolution and soft issue contrast than other models of assessment and has been recognized as the best non-invasive method for prostate examination [8–10]. T2-weighted imaging (T2WI) is a conventional MRI method that has been mature for nearly 20 years, but it seems that the sensitivity and specificity are not accurate as we expected. To improve the diagnostic accuracy of T2WI, multi-parametric MRI (mp-MRI) sequences such as dynamic contrast-enhanced (DCE) imaging, diffusion-weighted imaging (DWI), and magnetic resonance spectroscopy (MRS) have been recommended as adjuncts to conventional T2WI. These functional MRI techniques can provide metabolic information, display altered cellularity, and aid in the noninvasive characterization of tissue and tumor vascularity [11].

In 1971, Folkman [12] was the first to propose that tumor angiogenesis plays an important role in tumor development, invasion and metastasis across various stages. Other studies later showed that angiogenesis during the pathological staging of PCa could be used as a separate index to assess the formation and development of the disease [13]. DCE-MRI can be used to assess tissue and tumor vascular properties and is a rapidly evolving and noninvasive MRI technique. Although DCE is recommended by the European Society of Urogenital Radiology (ESUR) as a valuable functional technique in PCa detection [6], the accuracy of this method has seldom been systematically reviewed. Therefore, we performed a systematic review and meta-analysis of this technique to examine its diagnostic accuracy in the context of PCa.

## RESULTS

### Literature search

**Figure 1:**
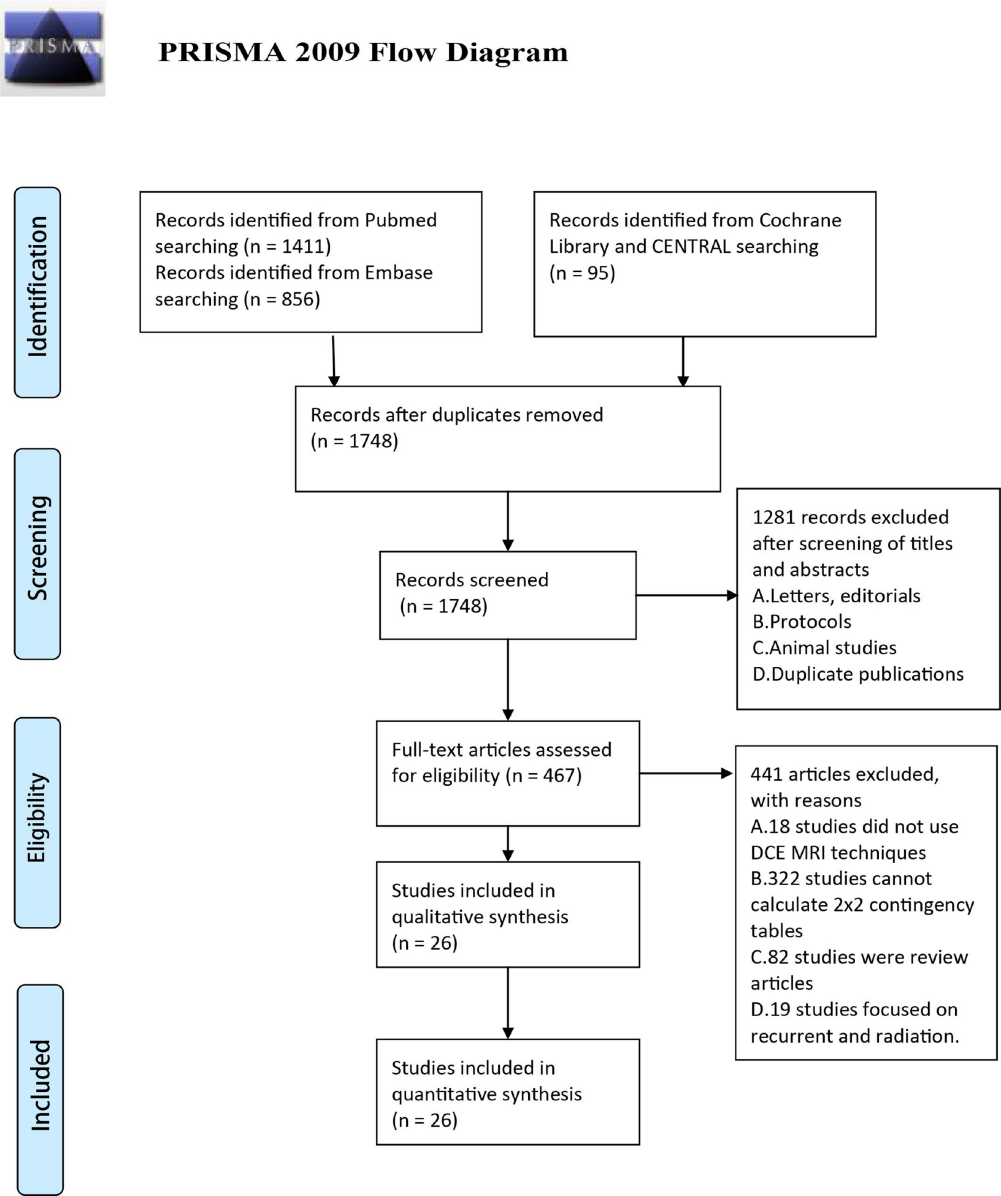
PRISMA 2009 flow diagram

Based on our comprehensive computerized search strategy, Figure 1 provides an overview of the literature search and study selection. Our search yielded 1411 unique records in PubMed,856 unique records in Embase and 95 unique records in Cochrane Library and CENTRAL. Of these, 1748 citations were assessed for eligibility, and the remaining 594 were rejected because they were duplicates. After reviewing the full articles, 441 studies were ultimately excluded for failing to meet the inclusion criteria. Articles were excluded if they did not use DCE-MRI techniques (*n* = 18); did not provide sufficient available data to construct a 2 × 2 table (*n* = 322); were review articles (*n* = 82) or studies focused on recurrent cancer and radiation (*n* = 19). These exclusions yielded a final set of 26 studies for inclusion in the systematic review [14–39]. There were no disagreements between the authors about the number of studies eligible for inclusion.

Of the 26 studies included in our review, two calculated per-regions and per-patients [26, 29]. We identified 21 studies that included DCE combined with other imaging techniques studies of PCa for localization of the tumor (peripheral zone, transition zone and whole gland [14–34], and 7 studies investigated each imaging technique performed per-patients analysis [26, 29, 35–39].

### Characteristics of the included studies

The selected studies included a total of 2070 patients. A total of 1163 patients were included in per-regions studies; 1055patients were included in per-patients studies. Patient age ranged from 39–88 years, and the average sample size across studies was 79 participants (range = 16–555). Across all studies, the PSA value ranged from 0.18–568.5 ng/ml.

Table 1 summarizes the patient, technical, and study characteristics.

**Table 1:** Summary of articles reviewed

Modality		No. of Articles (Whole gland/PZ/TZ)	No. of individuals (Whole gland/PZ/TZ)
By biopsy	DCE	12/6/2	8781 /3040/563
	DWI	8/5/1	7242/2285/1117
	DCE and DWI	2/1/0	2133/ 709/ND
	MRS	2/1/0	854/31/ ND
	T2	11/6/4	7187/3463/1558
	DCE and T2	2/0/1	1088/ND/42
	DWI and T2	1/0/2	424/ND/678
	DCE, DWI, and T2	7/1/3	4900/1134/1246
	DCE, DWI, MRS and T2	1 /0 /0	605/ND/ND
By patients	DCE	2	122
	DWI	2	120
	T2	3	145
	DCE and T2	2	598
	DWI and T2	2	90
	DCE, DWI, and T2	4	276
	DCE, DWI, MRS and T2	2	142
	T2 and MRS	1	70

Abbreviations: DCE: dynamic contrast-enhanced magnetic resonance; DWI: diffusion weighted imaging; T2: T2 weighted imaging; MRS: magnetic resonance spectroscopy; WR: whole region; PZ: peripheral zone; TZ: transition zone; ND: no data.

The characteristics of the included studies are illustrated separately in Table 2. Supplementary Table 1 shows individual study results from included studies.

**Table 2:** The characteristics of all the studies included

First author(year)	Patient characteristics						Study characteristics								Imaging characteristics		
	Age (yr)		PSA (ng/ml)		Gleason score		No.of patients	Reading (retrospective/prospective)	Patient enrollment	Analysis	Readers	Blinding	Prior prostate Bx	Reference test	Tesla	ERC	Study population
	Median or mean (*n*)	Range	Median or mean (*n*)	Range	Median or mean (*n*)	Range											
Abd-Alazeez (2013)	64 (Median)	39–75	10(Median)	4–23	NR	6–8	54	NR	NR	Biopsy	8	Y	TRUS Bx-	TPM	1.5T, 3T	N	Single center
Aydin (2012)	69 (mean)	54–82	70.6(Median)	1.6–139.53	7(mean)	5–10	40	prospective	consecutive	Biopsy	2	NR	NR	TRUS or RP	1.5T	N	Single center
Baur (2014)	66 (mean)	54–78	10(Median)	2.9–65.2	NR	6–10	55	retrospective	NR	Biopsy	2	NR	54 patients TRUS Bx-	MR-TRUS-fusion Bx	1.5T	N	Single center
Chabanova (2011)	64.18 (mean)	51–74	7(mean)	1.3–28	NR	4–9	43	NR	consecutive	Biopsy	2	Y	NR	TRUS	1.5T	Y	Single center
Iwazawa (2011)	68.8 (mean)	41–86	20.51(mean)	4.04–568.5	7.04(mean)	6–9	178	retrospective	consecutive	Biopsy	2	Y	NR	TRUS	1.5T	N	Single center
Kim CK (2006)	73 (mean)	58–71	16.9(mean)	0.6–55.4	NR	NR	20	NR	consecutive	Biopsy	2	NR	NR	TRUS	3T	N	Single center
Kim JK (2005)	64.9 (mean)	49–75	NR	2.6–43.5	NR	6–10	53	retrospective	NR	Biopsy	4	Y	NR	RP	1.5T	N	Single center
Kitajima (2010)	69 (Median)	56–84	11.1(Median)	4.2–112.1	NR	NR	53	retrospective	consecutive	Biopsy	2	Y	NR	TRUS	3T	Y	Single center
Kozlowski (2010)	61.7 (mean)	38–72	8.5(mean)	0.94–15	NR	6–9	25	prospective	consecutive	Biopsy	NR	NR	no prior treatment	MR-TRUS-guide Bx	3T	Y	Multicenter
Reisaeter (2015)	60.7 (mean)	42.9–70.3	11.6(mean)	3–81.4	NR	6–9	63	retrospective	consecutive	Biopsy	3	NR	NR	WMS	1.5T	Y	Single center
Portalez (2010)	62.4 (mean)	49–76	9.16(mean)	1.6–25	NR	NR	68	prospective	consecutive	Biopsy	1	NR	TRUS Bx-	TRUS	1.5T	Y	Single center
Puech (2009)	62 (mean)	46–76	8.15(mean)	1.45–26.4	NR	NR	83	NR	consecutive	Biopsy	2	NR	DRE+	WMS	1.5T	N	Single center
Rosenkrantz(2012)	63 (mean)	48–85	7.4(mean)	1–40	NR	5–7	42	retrospective	NR	Biopsy/ Patient	2	NR	29 patients TRUS Bx-	TRUS	3T	N	Single center
Rosenkrantz(2015)	62 (mean)	46–81	6.9(mean)	NR	NR	6–9	106	retrospective	NR	Biopsy	3	Y	NR	RP	3T	N	Single center
Tamada (2011)	70 (mean)	40–84	6.84(mean)	4.06–9.94	7(Median)	6–10	50	retrospective	NR	Biopsy/ Patient	2	Y	NR	TRUS	1.5T	N	Single center
Tamada (2008)	71 (mean)	62–84	16.9(mean)	2.98–125	7(Median)	5–10	40	retrospective	NR	Biopsy	2	NR	NR	TRUS	1.5T	N	Single center
Turkbey(2011)	60.2 (mean)	49–75	6.37(mean)	2.3–23.7	7(Median)	6–9	45	prospective	consecutive	Biopsy	2	NR	NR	WMS	3T	Y	Single center
Van den Bergh(2013)	65 (Median)	49–74	10.4(Median)	1.5–70.9	NR	6–10	73	prospective	consecutive	Biopsy	1	Y	NR	RP	1.5T	N	Single center
Weidner (2011)	63.5 (Median)	40–79	NR	4.25–137	NR	5–8	16	retrospective	NR	Biopsy	2	Y	NR	RP	1.5T	Y	Single center
Yoshizako(2008)	65 (Median)	52–76	NR	NR	NR	6–9	35	retrospective	NR	Biopsy	2	Y	NR	RP	1.5T	N	Single center
Yu(2008)	62.5 (Median)	46–75	11.2(mean)	5–39.1	NR	NR	21	retrospective	consecutive	Biopsy	2	Y	NR	RP	3T	N	Single center
Zhang(2014)	75 (mean)	50–83	14.19(median)	4.59–470.3	NR	7–9	75	NR	consecutive	Patient	2	Y	NR	TRUS	3T	N	Single center
Ferda(2013)	NR	47–79	NR	4.2–123	NR	NR	164	prospective	NR	Patient	NR	NR	NR	TRUS	3T	N	Single center
Vilanova(2011)	63.5 (mean)	43–87	7.4 (Median)	4–17.20	7(Median)	5–8	70	retrospective	NR	Patient	3	Y	NR	TRUS	1.5T	Y	Multicenter
Watanabe(2010)	72.2 (mean)	55–88	6.7(Median)	2.5–33.53	6.4(Mean)	4–9	43	retrospective	consecutive	Patient	1	NR	NR	TRUS/RP	1.5T	N	Multicenter
Haffner(2011)	64 (Median)	47–83	6.75(Median)	0.18–100	NR	NR	555	NR	consecutive	Patient	NR	NR	NR	TRUS	1.5T	N	Single center

Abbreviations: NR: not reported; ERC: endorectal coil; TRUS: transrectal ultrasound; Bx: biopsy; Bx-: negative TRUS-guided Bx; TPM: transperineal template prostate mapping; WMS: whole-mount section histopathology; DRE+: positive digital rectal examination; RP: radical prostatectomy.

### Methodological quality assessment

The majority of studies were considered to have a high risk of bias for the patient selection (57%, 15/26), index test (8%, 2/26). The 4 studies for which the risk of bias for patient selection was unclear did not report exclusion criteria or whether or not the sample was consecutive.

**Figure 2:**
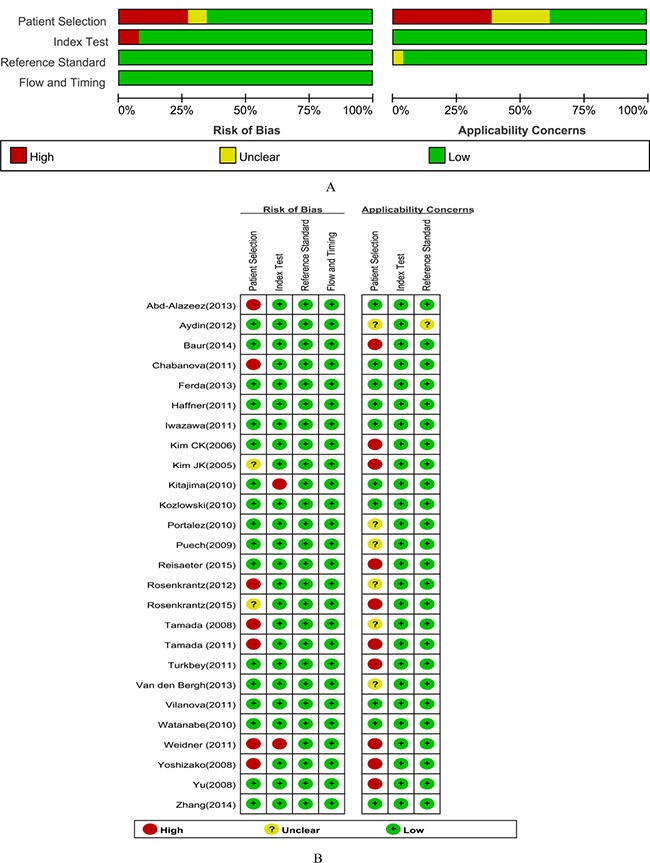
Methodological quality graph and summary

The results of the QUADAS-2 assessment are presented in Figure 2A–2B. All included studies fulfilled the 7 criteria of the QUADAS-2 regarding methodological quality.

### Investigations of heterogeneity

The heterogeneity tests of the sensitivity and specificity values were calculated, respectively. Highly significant heterogeneity was detected when all of the studies were pooled. We chose a random-effect model provided by the software. A single-factor meta-regression analysis and the subgroup analysis contributed significantly to this heterogeneity.

### Publication bias

The non-significant slope of Deeks’ funnel plot asymmetry test (*P* > 0.1) indicates the absence of publication bias. No significant publication bias was observed for the whole gland (*P* = 0.91) and peripheral zone (*P* = 0.87).

### Biopsy-level analysis of diagnostic performance summary estimates

**Figure 3:**
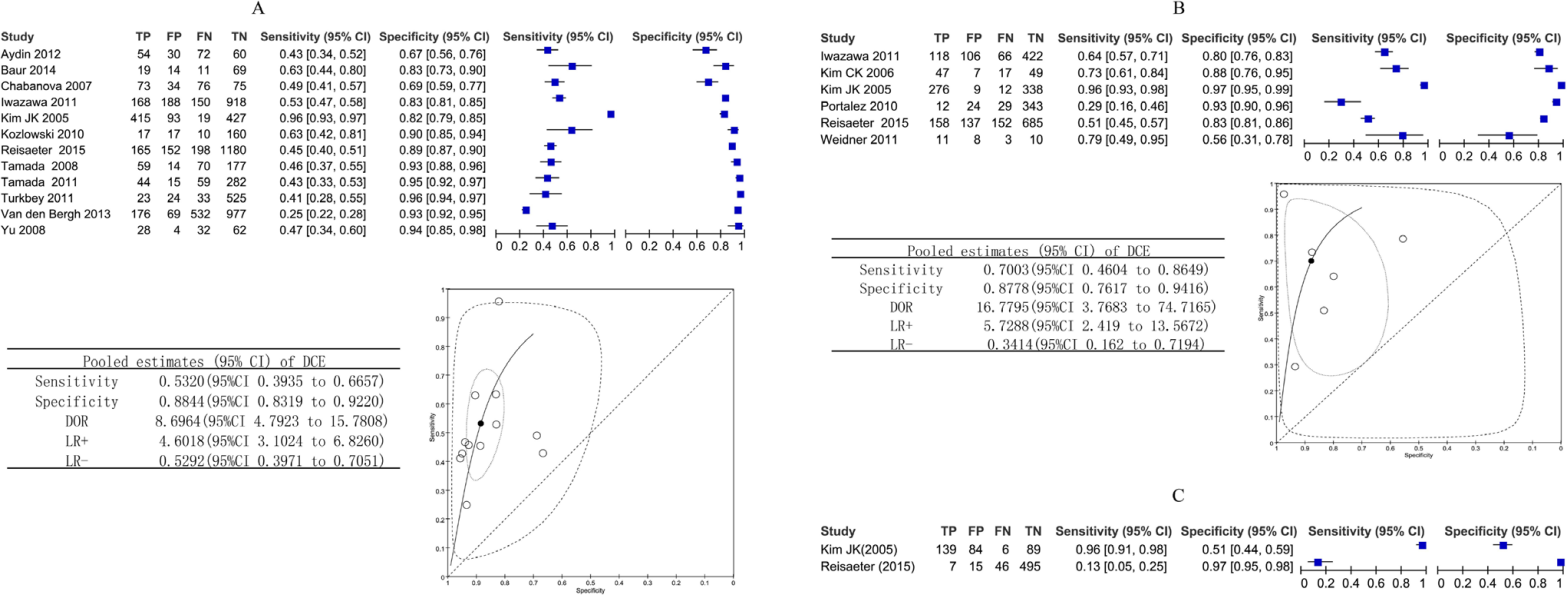
(**A–C**) Biopsy-level analysis of DCE. Dynamic contrast-enhanced MRI biopsy-level analysis: forest plots, pooled estimates and SROC curve showed in whole gland (A) and peripheral zone (B), forest plot showed in transition zone (C).

DCE-MRI was compared on the whole gland in 12 studies that provided sufficient information for inclusion in a meta-analysis. The sensitivity varied from 0.25 to 0.96, and the specificity ranged from 0.67 to 0.96. The pooled sensitivity was 0.53 (95% CI 0.39 to 0.67), and the pooled specificity was 0.88 (95% CI 0.83 to 0.92). Overall, DCE had a diagnostic odds ratio (DOR) of 8.69 (95% CI 4.79 to 15.78) and an area under the curve (AUC) of 0.84 (95% CI 0.80 to 0.87). Figure 3A shows the sensitivity and specificity of the individual studies, pooled estimates and SROC curve.

The diagnostic accuracy of DCE-MRI in the peripheral zone was reported in 6 studies, and the sensitivity estimates and specificity ranged from 0.29 to 0.96 and 0.56 to 0.97. The pooled (95% CI) estimates for sensitivity and specificity were 0.70 (95% CI 0.46 to 0.86) and 0.88 (95% CI 0.76 to 0.94), respectively. DOR was 16.7 (95% CI 3.77 to 74.72), and the AUC was 0.88 (95% CI 0.85 to 0.91). Figure 3B shows the sensitivity and specificity of the individual studies, pooled estimates and SROC curve.

Two studies reported the results of transition zone at biopsy of DCE-MRI. In the study by Kim JK *et al.* [20], which enrolled 53 participants, the sensitivity was 0.96 (95% CI 0.91 to 0.98), and the specificity was 0.51 (95% CI 0.44 to 0.59). In the study by Reisaeter *et al.* [23], which enrolled 63 participants, the sensitivity was 0.13 (95% CI 0.05 to 0.25), and the specificity was 0.97 (95% CI 0.95 to 0.98). Figure 3C shows the sensitivity and specificity of the individual studies.

### Studies directly comparing tests

### Comparative analysis: DCE versus DWI

For the whole gland, eight studies involving 544 patients reported DCE compared with DWI and provided sufficient information for inclusion in a meta-analysis. DWI appeared to have a statistically higher pooled sensitivity (*P* < 0.001); the pooled estimates for sensitivity and specificity were 0.43 (95% CI 0.36 to 0.51) and 0.90 (95% CI 0.84 to 0.94) for DCE and 0.54 (95% CI 0.42 to 0.67) and 0.89 (95% CI 0.83 to 0.92) for DWI.

When we included four studies of the peripheral zone involving 325 patients that reported DCE compared with DWI, DWI appeared to have a statistically higher pooled sensitivity (*P* = 0.0498). The pooled estimates for sensitivity and specificity were 0.56 (95% CI 0.35 to 0.74) and 0.82 (95% CI 0.69 to 0.91) for DCE and 0.67 (95% CI 0.45 to 0.84) and 0.81 (95% CI 0.57 to 0.93) for DWI.

For the transition zone, Reisaeter *et al.* [23] in a study of 63 patients reported DCE compared with DWI. DCE and DWI appeared to have similar sensitivity, respectively, with values of 0.13 (95% CI 0.05 to 0.25) versus 0.17 (95% CI 0.08 to 0.29), and specificity, with values of 0.97 (95% CI 0.95 to 0.98) versus 0.94 (95% CI 0.91 to 0.96), respectively.

**Figure 4:**
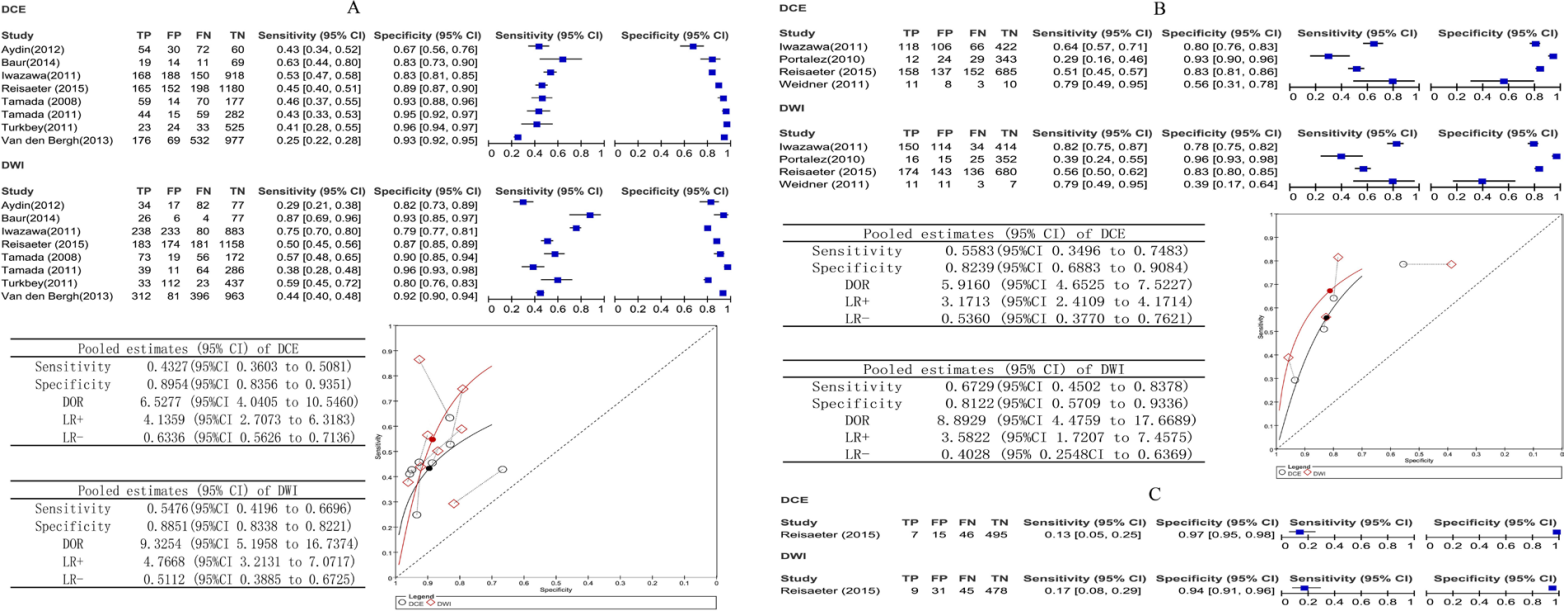
(**A–C**) Comparative analysis DCE versus DWI. Dynamic contrast-enhanced MRI compared with DWI biopsy-level analysis: forest plots, pooled estimates and SROC curve showed in whole gland (A) and peripheral zone (B), forest plot showed in transition zone (C).

Figure 4A–4C shows the sensitivity and specificity of the individual studies, pooled estimates and SROC plot with the 95% confidence region.

### Comparative analysis: DCE versus T2

For the whole gland, ten studies involving 483 patients reported DCE compared with T2 and provided sufficient information for inclusion in a meta-analysis. There was evidence that DCE had better test accuracy than T2. Using the results from the earlier analysis, DCE appeared to have higher pooled sensitivity (*P* = 0.1666) and statistically higher specificity (*P* < 0.001). The pooled estimates for sensitivity and specificity were 0.52 (95% CI 0.36 to 0.68) and 0.89 (95% CI 0.82 to 0.93) for DCE and 0.50 (95% CI 0.41 to 0.58) and 0.85 (95% CI 0.75 to 0.91) for T2.

For the peripheral zone, five studies involving 220 patients reported DCE compared with T2. The pooled estimates for sensitivity and specificity were 0.71 (95% CI 0.42 to 0.89) and 0.89 (95% CI 0.75 to 0.95) for DCE and 0.59 (95% CI 0.46 to 0.72) and 0.73(95% CI 0.57 to 0.84) for T2. These results indicate that DCE had statistically higher sensitivity (*P* = 0.0194) and specificity (*P* = 0.006) in the peripheral zone compared with T2.

For the transition zone, two studies involving 116 patients reported DCE compared with T2. In Reisaeter *et al.* [23], the sensitivity was 0.13 (95% CI 0.05 to 0.25), and the specificity was 0.97 (95% CI 0.95 to 0.98) for DCE, whereas the sensitivity was 0.13 (95% CI 0.05 to 0.25), and the specificity was 0.94 (95% CI 0.92 to 0.96) for T2. In Kim JK *et al.* [20], the sensitivity was 0.96 (95% CI 0.91 to 0.98), and the specificity was 0.51 (95% CI 0.44 to 0.59) for DCE, whereas the sensitivity was 0.45 (95% CI 0.37 to 0.98), and the specificity was 0.73 (95% CI 0.66 to 0.79) for T2.

**Figure 5:**
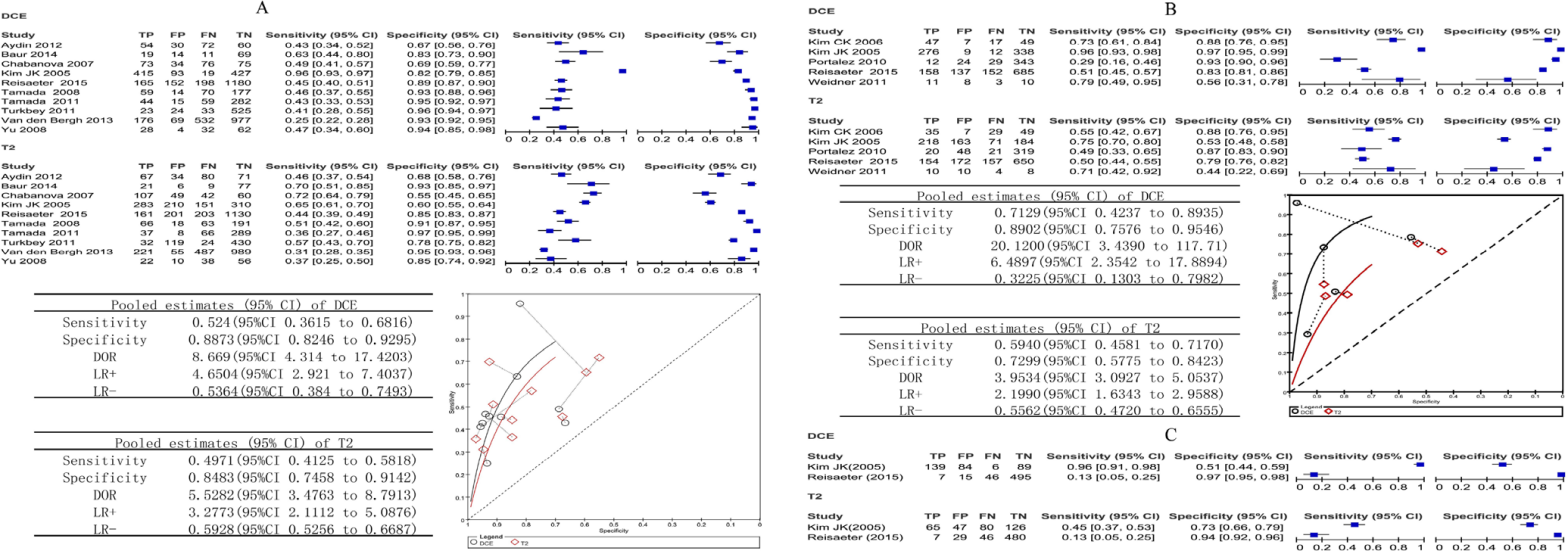
(**A–C**) Comparative analysis DCE versus T2. Dynamic contrast-enhanced MRI compared with T2 biopsy-level analysis: forest plots, pooled estimates and SROC curve showed in whole gland (A) and peripheral zone (B), forest plot showed in transition zone (C).

Figure 5A–5C shows the sensitivity and specificity of the individual studies, pooled estimates and SROC plot with the 95% confidence region.

### Comparative analysis: DCE versusT2 combined with DCE and DWI

For the whole gland, five studies involving 281 patients reported DCE compared with DCE versus T2 combined with DCE and DWI and provided sufficient information for inclusion in a meta-analysis. The pooled estimates for sensitivity and specificity were 0.41 (95% CI 0.33 to 0.52) and 0.92 (95% CI 0.89 to 0.95) for DCE, and 0.61 (95% CI 0.53 to 0.68) and 0.90 (95% CI 0.85 to 0.93) for T2 combined with DCE and DWI, it shows statistically difference in sensitivity (*p* < 0.0001).

For the peripheral zone, Reisaeter *et al.* [23] reported in a study involving 63 patients. T2 combined with DCE and DWI appeared to have slightly higher sensitivity 0.60 (95% CI 0.55 to 0.66) versus DCE 0.51 (95% CI 0.45 to 0.57) and similar specificity.

For the transition zone, Reisaeter *et al.* [23] reported DCE compared with T2 combined with DCE and DWI. The sensitivity and specificity were 0.13 (95% CI 0.05 to 0.25) and 0.97 (95% CI 0.95 to 0.98) for DCE and 0.15 (95% CI 0.07to 0.27) and 0.95 (95% CI 0.93 to 0.97) for T2 combined with DCE and DWI. Thus, T2 combined with DCE and DWI appeared to have slightly higher sensitivity.

**Figure 6:**
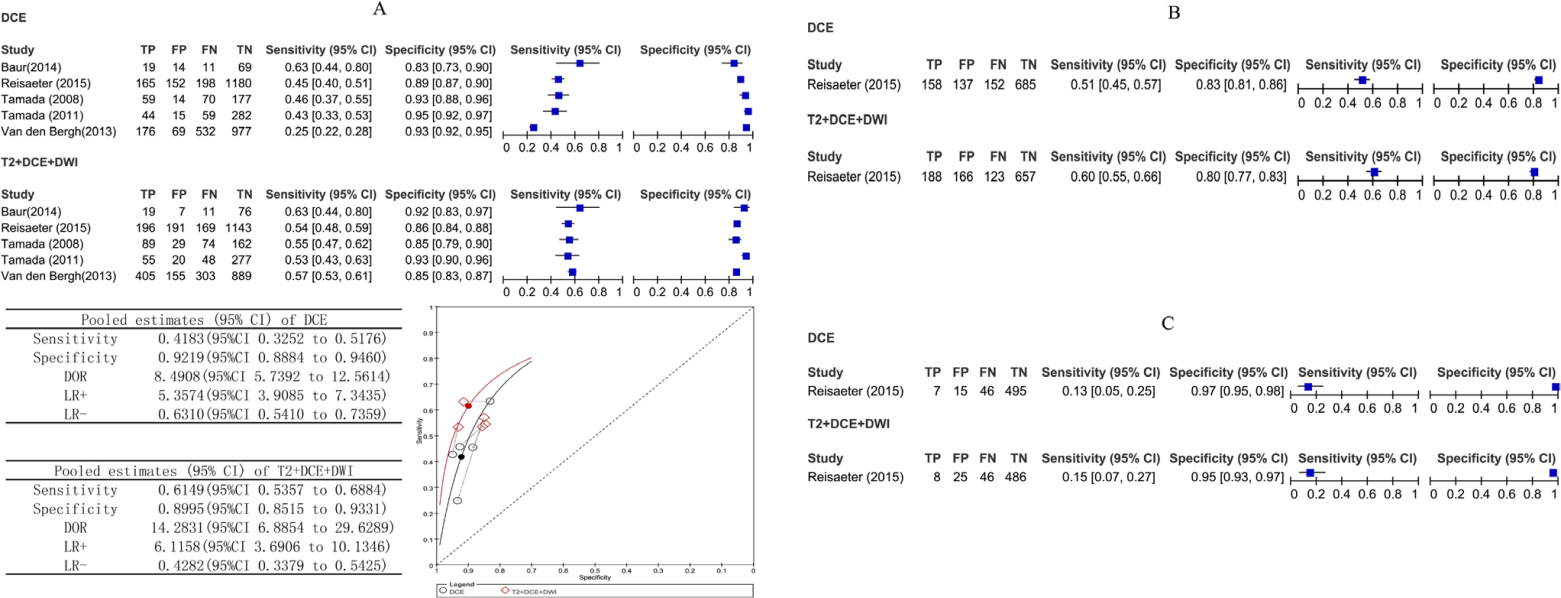
(**A–C**) Comparative analysis DCE versus T2 combine DCE and DWI. Dynamic contrast-enhanced MRI compared with T2 combine DCE and DWI biopsy-level analysis: forest plot, pooled estimates and SROC curve showed in whole gland (A), forest plots showed in peripheral zone (B) and in transition zone (C).

Figure 6A–6C shows the sensitivity and specificity of the individual studies, pooled estimates and SROC plot with the 95% confidence region.

### Other direct comparison tests

To assess other direct comparison tests, data were derived from only one or two studies. Because of the clear evidence of incorporation bias, we did not perform a meta-analysis of measures of test accuracy.

Aydin [15], Turkbey [30] and Weidner [32] directly compared DCE and MRS. For the whole gland, Aydin [15] reported DCE slightly higher specificity and lower sensitivity compared to MRS, whereas Turkbey [30] reported the slightly lower specificity and higher sensitivity compared to MRS. For the peripheral zone, MRS had slightly higher specificity and similar sensitivity. Iwazawa [18] showed that DCE combined with DWI had significantly higher sensitivity than DCE.

Supplementary Table 2 shows the results of the other direct comparison as above.

### Studies indirectly comparing tests for the biopsy-level pooled estimates

For the biopsy-level estimates of tests with four or more studies, compared with DWI, DCE was observed to have equivalent sensitivity and specificity on whole gland, and have higher sensitivity, specificity and diagnosis accuracy than T2.

Supplementary Table 3 shows the results for the biopsy-level pooled estimates from indirect comparisons.

### Studies for patient-level analysis

There was none of test included in four or more studies. So we carried out no meta-analyses due to insufficient data for pooling on any outcome. therefore, we reported outcomes narratively and summarised in tabular format.

Supplementary Table 4 shows the results for the patient-level analysis.

### Subgroup analysis

Significant heterogeneity were observed, As we used subgroup and meta-regression analysis that may conducted with regard to different study characteristics. The source of heterogeneity mainly revealed the method of DCE technology (*p* = 0.0093). but we should not neglected that the heterogeneity due to the heterogeneous nature of the related studies and inevitable in clinical practice. The subgroup analyses showed the studies by using quantitative methods and 3.0 Tesla had higher sensitivity and specificity.

Table 3 contains results of this subgroup analysis.

**Table 3:** Subgroup analysis and meta-analysis

Study characteristics	No	sensitivity	specificity	DOR	Chi2 test of heterogeneity	*P* value for heterogeneity	*P-*value
Total	12	0.53 (0.39–0.67)	0.88 (0.83–0.92)	8.70 (4.80.-15.78)	181.08		
Field strength							0.2326
1.5T	9	0.54 (0.39–0.70)	0.86 (0.81–0.91)	7.34 (3.81–14.15)	170.72	< 0.001	
3.0T	3	0.50 (0.22–0.79)	0.94 (0.89–0.99)	15.9 (10.27–24.62)	0.05	0.947	
Coil							0.796
Without endorectal coil	8	0.55 (0.38–0.72)	0.88 (0.83–0.94)	10.11 (4.68–21.85)	153.44	< 0.001	
With endorectal coil	4	0.49 (0.25–0.73)	0.89 (0.81–0.96)	7.22 (3.18–16.37)	28.02	< 0.001	
Blind							0.5910
Unblind	5	0.51 (0.29–0.73)	0.87 (0.80–0.95)	8 (3.87–16.55)	35.20	< 0.001	
Blind	7	0.55 (0.37–0.73)	0.89 (0.84–0.94)	10.10 (4.24–24.03)	145.55	< 0.001	
Patient enrollment							0.1051
Non-consecutive or unclear	4	0.69 (0.50–0.88)	0.90 (0.83–0.96)	19.37 (5.49–68.36)	43.68	< 0.001	
consecutive	8	0.45 (0.30–0.60)	0.88 (0.82–0.93)	6.12 (3.98–9.39)	52.13	< 0.001	
Methods							0.0093
Traditional methods	5	0.36 (0.33–0.39)	0.89 (0.88–0.90)	8.62 (5.6–12.26)	14.71	0.005	
Semi-parametric methods	3	0.48 (0.42–0.54)	0.72 (0.67–0.77)	2.75 (1.20–6.57)	9.77	0.008	
parametric methods	4	0.68 (0.49–0.88)	0.90 (0.84–0.96)	19.92 (4.63–85.61)	91.99	< 0.001	

Abbreviations: DOR: diagnostic odds ratio.

## DISCUSSION

There are different opinions regarding the added value of DCE sequences. For example, according to the new PI-RADS v2 guideline [40], DCE-MRI technology is an essential component of the mp-MRI prostate examination. However, DCE is not recommended as a mp-MRI approach for PCa detection by the National Institute for Health and Care Excellence (NICE) guidelines [41].

In this study, it was showed that the specificity values for detection PCa on both whole gland and PZ were approximately 0.88, indicating that DCE-MRI can distinguish between normal tissues from PCa. Biger *et al.* demonstrated that PCa was associated with an approximately two-fold increase in the total number of vessels seen on histologic sections [42]. Based on the findings from Biger *et al.*, Engelbrecht *et al.* reported that PCa showed more pronounced enhancement resulting in higher signal on DCE-MRI than surrounding normal prostate tissues [43]. Therefore, DCE-MRI shows its ability in the localizing of PCa. Moreover, it has high accuracy in detecting cancer recurrence who have undergone radical prostatectomy [44] or post-radiotherapy patients [45]. Meanwhile, our direct comparison study showed that the specificity of DCE was statistically superior to T2WI ( 0.89 vs 0.73, *p* < 0.0001). The study by Shimizu *et al.* also showed that low specificity (0.70) and sensitivity (0.29) limited the use of T2WI [46]. T2WI was a standard sequence in detecting PCa, in which tumor tissue appears hypointense relative to the normal peripheral tissue [47]. However, focal decreases in T2 signal intensity can result from benign processes and PCa may also show minimally reduced T2 signal, making them nearly isointense on T2WI [48]. The reports from Rosenkrantz *et al.* suggested that DCE sequences were mainly effective in detecting non-nodular, infiltrating vascular lesions, which were poorly visible on T2WI or DWI [49].

However, the sensitivity value of DCE-MRI for locating PCa was relatively low on the whole gland (0.55) and PZ (0.70), suggesting that DCE-MRI had difficulty in distinguishing between malignant tumors and benign lesions. Agha *et al.* reported that some benign prostatic lesions, such as benign prostatic hyperplasia (BPH), may show enhancement pattern of nearby criteria to the PCa enhancement curves, and some unavoidable technical errors, such as rectal motions, may distort the relatively long timed dynamic sequences [50]. Although Tofts *et al.* [51] standardized acquisition parameters of quantitative analysis, there is also a problem of the rate constants overlapping between benign and malignant tissues [52]. Meanwhile, our direct comparison reflected that the sensitivity values of DCE were statistically lower on whole gland and relatively lower on PZ than the values from DWI. This could be explained at least in part by different approaches to evaluate DCE-MRI in a qualitative or quantitative way [16]. However, Iwazawa *et al.* suggested that the prostate lesions were also missed when detection was attempted by DWI alone. Thus, excluding DCE from routine prostate MRI incurs a risk of failure in the detection of PCa, especially for lesions in the PZ [18].

By our subgroup and meta-regression analysis, it was found that the main source of heterogeneity was from the method of DCE technology. Compared to the qualitative and the semi-parametric method, parametric methods have a slight improvement in the sensitivity and DOR. Qualitative DCE-MRI is based on the subjective evaluation by the experience of the observer and semi-parametric uses signal intensity time curves (SI) to classify the tissue. Both qualitative and semi-parametric methods directly or indirectly focus on evaluating the strength of enhancement of the regions of interest (ROIs) [53]. However, based more on simulating the change in the concentration agent using eligible pharmacokinetic (PK) modeling techniques, the parametric method can determine the rate of contrast exchange and derived quantitative modeling parameters such as Ktrans and Kep [54]. This method, consistent with the theory by Folkman [12], not only further computerizes empirical parameters, but also reduces the bias among different observers.

Finally, we acknowledge certain limitations of this meta-analysis. Firstly, TZ tumors are estimated to account for approximately 30% of PCa cases and pose a clinical challenge because of the difficulty of their detection [55] despite compelling data supporting the value of MRI for improving PZ tumor detection [56]. The actual clinical utility of MRI for improving TZ tumor detection was still unclear. Sinnott *et al.* inferred that different prostate zones could point a key source of variability in PCa prognosis and treatment response [57]. Therefore, PZ, TZ, and whole gland should not be combined in a single test due to the different incidence of the disease, which may have an impact on the diagnostic performance. Villers *et al.* found that DCE-MRI was an accurate technique for detecting and quantifying TZ tumor with early enhancement which had no suspicious on T2WI [58]. However, Hoeks *et al.* reported that DWI and DCE may not improve TZ cancer detection and localization accuracy compared with T2WI [59]. Our study had included 4 literatures [20, 23, 27, 33], which had focused on TZ, but it was insufficient for a meaningful pooling estimate results. Therefore, the effects on TZ tumor detection need to be clarified with more literatures in the near future.

Secondly, according to the method by Cheikh *et al.*, who found that “per-region” and “per-patient” analysis may have a dramatic difference in localizing PCa [60], we prospectively designed the ‘per-patient’ analysis. The sensitivity and specificity showed dramatic differences compared to ‘per-region’ analysis across the included studies. Our results were consistent with Cheikh *et al.*, who suggested that ‘per-region’ analysis could lead to an overestimation of specificity due to high proportion of regions without cancer. In our analysis, there was insufficient data for pooling results and the meaningful pooling results need more research support.

Lastly, most studies used a combination of targeted and systematic biopsies. Although biopsies have been recommended as an gold standard in detected PCa in several guidelines [61–62], it might tend to miss tumors on first systematic biopsy [63], and the diagnostic accuracy might therefore be slightly interfered. Some of the individual studies had limited quality, which may influenced our meta-analysis outcomes.

## MATERIALS AND METHODS

### Study registration

The protocol was registered at the international prospective register of systematic reviews (PROSPERO) website under number CRD42017056236 [64]. This study followed the PRISMA statement and the Cochrane Handbook for Diagnostic Test Accuracy Reviews [65–66].

Institutional review board ethical approval was not needed because of the reviewing nature of this study.

### Criteria for considering studies for this review

### Type of studies

We first included a study with sufficient data to evaluate the diagnostic accuracy of DCE-MRI for the diagnosis of PCa using histopathological assessment as the reference standard.

We also performed direct and indirect comparisons with other MRI methods and to explore the strengths and shortcomings of DCE-MRI [67].

### Participants

We included adult patients with clinical symptoms suspicious for PCa who underwent DCE-MRI examination and biopsy.

### Index test

Studies that assessed the accuracy of diagnostic tests included DCE with or without other methods of imaging such as MRS, T2WI or DWI alone or in combination were included.

### Reference standards

The reference standard was histopathological assessment of biopsied tissue. Tissue samples were obtained by surgery, autopsy, or TRUS-guided biopsy. The units of analyses reported by the studies included biopsy, site, segment, region and core.

### Types of outcomes

The primary objective of our study is to evaluate the diagnostic capability of DCE-MRI in patients suspected of having PCa. A secondary outcome is to compare DCE with other MRI methods such as DWI, T2WI, MRS to explore the strengths and shortcomings of DCE.

### Literature search strategy

Two reviewers performed a comprehensive literature search to identify relevant studies published in English. An electronic search of articles published from January 2000 to September 2016 was performed using PubMed and Embase by incorporating the following keywords: “prostatic OR prostate tumor OR prostate cancer OR prostate carcinoma” AND “magnetic resonance imaging OR MRI OR magnetic resonance” AND “contrast OR contrast medium OR DCE ORDCE-MRI OR dynamic OR contrast enhance”. Other sources such as the Cochrane Library were also checked for relevant studies using similar keywords. Abstracts were reviewed for relevance to the defined review question. If it was not clear from the abstract whether the paper might contain relevant data, the full paper was assessed.

Supplementary Table 5 shows the search strategies for the searches.

### Study selection and data extraction

Two reviewers independently conducted the study selection protocol. Articles that were not excluded by at least one investigator were scrutinized by checking the full text independently. Final inclusion was determined after discussion to resolve any discrepancies. Duplicate use of the same data was carefully excluded.

A study was included for review if it met the following inclusion criteria: A. the research focused on the preoperative diagnosis of prostate cancer; B. DCE-MRI was performed with a gadolinium agent and used to identify and characterize prostate cancer; C. the study incorporated the ‘gold standard’ of histopathological analysis performed during surgery or autopsy, or a TRUS-guided biopsy was used as the reference standard; and D. sufficient data were presented directly or indirectly to calculate 2 × 2 contingency tables for per-patient or per-lesion statistics.

The following exclusion criteria were also applied: A. all review articles, letters, comments, case reports and non-clinical trials were eliminated from consideration; B. studies with fewer than 10 patients in the sample were excluded; C. Studies featuring patients with previous recurrences and who have received radiation therapy for prostate cancer will be excluded.

To conduct a reliable analysis, the following data were extracted: patient age, number of previous prostate biopsies, magnetic field strength (in Tesla), use of an endorectal coil, use of other coils, DCE-MRI acquisition parameters, use of additional techniques, year of publication, study population, reference standard (i.e. prostate biopsy or prostatectomy specimen), patient enrollment, study design, blinding, number of readers.

### Quality assessment

The same two review authors independently assessed the relevant extracted data. Any disagreements between the two reviewers were resolved by a third reviewer. The two reviewers each used 7 items from the published quality assessment of diagnostic accuracy studies version 2 (QUADAS-2) guidelines to assess the relevant studies. The QUADAS-2 tool is structured in a series of questions that should be answered ‘yes’, ‘no’, or ‘unclear’ and aims to evaluate study quality involving the participant spectrum, index test, reference standard, and flow and timing as ‘high risk’ ‘low risk’ and ‘unclear’. As a general rule, if a particular point was not mentioned in a document, then the relevant item of signaling was marked as ‘unclear’. If at least one answer to the signaling questions was ‘no’ or at least two answer ‘unclear’ of the four domains and applicability, we classified the studies as high risk of bias [68].

We summarized the methodological quality using the risk of bias and applicability concerns. Supplementary Table 6 shows the adopted items that served the purposes of our review.

### Data synthesis and analysis

The numbers of true positive (TP), false positive (FP), false negative (FN) and true negative (TN) findings with regard to MRI used to diagnose preoperative PCa were calculated from the included studies to construct 2 × 2 Tables.

We used data from the 2 × 2 tables to calculate sensitivity, specificity, positive likelihood ratios (LR+), and negative likelihood ratios (LR-) with 95% CIs and accuracy for each study. A continuity correction of 0.5 was added to all cells for studies that contained a count of zero to avoid potential problems with odds calculations for studies with sensitivity or specificity values of 100%.

The diagnostic odds ratio (DOR) was estimated based on the LR+ and LR− to represent the odds of a positive test among patients with PCa compared with the odds of a positive image among patients without PCa. This single indicator of test accuracy included a combination of sensitivity and specificity information [69]. We also plotted the sensitivity and specificity values to construct a summary receiver operating characteristic (SROC) curve. Then, we calculated the area under the SROC curve (AUC).

The directly comparative analysis was performed between all tests with four or more studies with relevant data. we compared the sensitivity and specificity of the direct comparison method to whether the t-statistics in the output provide statistically significant [70]. The comparative analysis consisted of an indirect comparison in which all tests with relevant data were compared and accuracy assuming a common underlying shape.

### Investigations of heterogeneity

Heterogeneity in meta-analysis refers to the degree of variability in study results. If heterogeneity existed, a random effect model was used for the primary meta-analysis to obtain a summary estimate for sensitivity with 95% CIs. An exploration of the reasons for heterogeneity rather than computation of a single summary measure is an important goal of meta-analysis.

### Publication bias

Publication bias was studied using Deeks funnel plots. The funnel plots was assessed visually by using a scatter plot of the inverse of the square root of the effective sample size (ESS)—1/ESS^1/2^ —versus the diagnostic log odds ratio (lnDOR), Formal testing for publication bias was conducted by using a regression of lnDOR against 1/ESS^1/2^ and weighting according to the ESS, with *P* < 0.10 indicating significant asymmetry [71].

### Subgroup analysis

If heterogeneity is detected, We using subgroup analysis and meta-regression to investigate the source of heterogeneity. subgroup analyses were performed by the study characteristics such as magnetic field strength, coils, reference standards, patient enrollment, study design, and blinding, the method of DCE technology. Meta-regression analysis was also performed to identify potential factors that could explain the source of heterogeneity [72]

### Software

The tables were entered into RevMan5 software (The Cochrane Collaboration, The Nordic Cochrane Centre, Copenhagen, Denmark) and SAS version 9.3 (SAS Institute Inc., Cary, NC, USA). All statistical analyses and graphical plots were performed in RevMan5.

## CONCLUSIONS

Our meta-analysis demonstrates that although DCE-MRI can provide informative supplementary diagnostic accuracy to detect PCa, it remains a confirmatory tool. As new quantitative techniques are developed to enhance the standards of optimal scans, DCE-MRI may attain important clinical status.

## SUPPLEMENTARY MATERIALS FIGURES AND TABLES








