# Supplementary material for: Accuracy of dynamic contrast-enhanced magnetic resonance imaging in the diagnosis of prostate cancer: systematic review and meta-analysis
Source: Oncotarget. 2017 Aug 17;8(44):77975–89. doi: 10.18632/oncotarget.20316 (PMC5652829; doi:10.18632/oncotarget.20316)
Supplement: Supplementary file 1 [file oncotarget-08-77975-s001.pdf]

Accuracy of dynamic contrast-enhanced magnetic resonance imaging in the diagnosis of prostate cancer: systematic review and meta-analysis

SUPPLEMENTARY MATERIALS

Supplementary Table 1: Individual study results. See_Supplementary Table_1

Supplementary Table 2: Other directly comparing tests

Comparative analysis	Region	Study	Test	No.analyzed	Sensitivity	Specificity
DCE versus MRS	whole gland	Aydin 2012	DCE	216	0.43	0.67
			MRS	246	0.69	0.49
	whole gland	Turkbey 2011	DCE	605	0.59	0.80
			MRS	608	0.29	0.98
	PZ	Weidner 2011	DCE	32	0.79	0.56
			MRS	31	0.79	0.76
DCE versus T2+DCE+DWI+MRS	whole gland	Turkbey 2011	DCE	605	0.59	0.80
			T2+DCE+DWI+MRS	605	0.61	0.73
DCE versus DCE+DWI	whole gland	Iwazawa 2011	DCE	1424	0.53	0.83
			DCE+DWI	1424	0.73	0.80
	PZ	Iwazawa 2011	DCE	712	0.64	0.80
			DCE+DWI	709	0.82	0.78

Abbreviations:
DCE: dynamic contrast-enhanced magnetic resonance. DWI: diffusion weighted imaging. T2: T2 weighted imaging. MRS: magnetic resonance spectroscopy. PZ:peripheral zone.

Supplementary Table 3: Biopsy-level pooled estimates from indirect comparison

Region	Test	No.studies	No.analyzed	Pooled estimates (95% CI)		AUC*
				Sensitivity	Specificity	
whole region	DCE	12	8189	0.53 [0.39–0.67]	0.88 [0.83–0.92]	0.84 [0.81, 0.87]
	DWI	8	6530	0.55 [0.42, 0.67]	0.89 [0.83, 0.92]	0.84 [0.80, 0.87]
	T2	11	7037	0.51 [0.43, 0.59]	0.86 [0.78, 0.91]	0.69 [0.65, 0.73]
	MRS	2	854	NA[1]	NA[1]	N^{A}1
	DCE+DWI	1	1424	NA[1]	NA[1]	N^{A}1
	T2+DCE	2	1088	NA[1]	NA[1]	NA[1]
	T2+DWI	1	424	NA[1]	NA[1]	NA[1]
	T2+DCE+DWI	8	4900	0.65 [0.57, 0.72]	0.86 [0.76, 0.92]	0.78 [0.74, 0.81]
	T2+DCE+DWI+MRS	1	605	NA[1]	NA[1]	NA[1]
PZ	DCE	6	2920	0.69 [0.40, 0.88]	0.88 [0.73, 0.95]	0.88 [0.85, 0.90]
	DWI	4	2285	0.67 [0.45, 0.84]	0.81 [0.57, 0.93]	0.80 [0.76, 0.83]
	T2	5	2209	0.59 [0.46, 0.72]	0.73 [0.58, 0.84]	0.73 [0.69, 0.77]
	MRS	1	31	NA[1]	NA[1]	N^{A}1
	DCE+DWI	1	709	NA[1]	NA[1]	NA[1]
	T2+DWI+DCE	1	1134	NA[1]	NA[1]	NA[1]
TZ	DCE	2	678	NA[1]	NA[1]	NA[1]
	DWI	1	563	NA[1]	NA[1]	NA[1]
	T2	4	1558	0.36 [0.21, 0.55]	0.89 [0.71, 0.96]	0.64 [0.60, 0.68]
	T2+DCE	1	42	NA[1]	NA[1]	NA[1]
	T2+DWI	2	678	NA[1]	NA[1]	NA[1]
	T2+DWI+DCE	3	1246	0.51 [0.23, 0.80]	0.97 [0.95, 0.98]	NA[1]

Abbreviations:
DCE: dynamic contrast-enhanced magnetic resonance. DWI: diffusion weighted imaging. T2: T2 weighted imaging. MRS: magnetic resonance spectroscopy. PZ: peripheral zone. TZ: transition zone. NA not available
1. Insufficient data for pooling results.

Supplementary Table 4: Patient-level analysis

First author (year)[ID]	No.Analyzed	Test	Sensitivity	Specificity
Rosenkrantz (2012) [26]	42	T2+DWI+DCE	1.00	0.74
Tamada (2011) [29]	50	DCE	0.74	0.8
	50	DWI	0.69	0.87
	50	T2	0.60	0.87
Zhang (2014) [35]	72	DCE	0.91	0.75
	75	T2	0.64	0.58
Ferda (2013) [36]	164	T2+DWI+DCE	0.98	0.65
Vilanova (2011) [37]	70	DWI	0.82	0.78
	70	T2+DWI	0.82	0.78
	70	T2+DWI+DCE+MRS	0.95	0.81
	70	T2+MRS	0.79	0.81
Watanabe (2010) [38]	43	T2+DCE	0.88	0.88
Haffner (2011) [39]	555	T2+DCE	0.83	0.61

Abbreviations:
DCE: dynamic contrast-enhanced magnetic resonance. DWI: diffusion weighted imaging. T2: T2 weighted imaging. MRS: magnetic resonance spectroscopy.

Supplementary Table 5: Search strategies. See_Supplementary Table_5

Supplementary Table 6: Quality assessment of diagnostic accuracy studies (QUADAS-2) checklist. See_ Supplementary Table_6
