# Supplementary material for: Accuracy of dynamic contrast-enhanced magnetic resonance imaging in the diagnosis of prostate cancer: systematic review and meta-analysis
Source: Oncotarget. 2017 Aug 17;8(44):77975–89. doi: 10.18632/oncotarget.20316 (PMC5652829; doi:10.18632/oncotarget.20316)
Supplement: Supplementary file 2 [file oncotarget-08-77975-s002.docx]

Supplementary Table 1: Individual study results

|  |  |  |  |  |  |  |  |  |  |
| --- | --- | --- | --- | --- | --- | --- | --- | --- | --- |
| First author (year)[ID] | Unit of analysis | Test | No. Analyzed | TP | FP | FN | TN | Sensitivity | Specificity |
|  |  |  |  |  |  |  |  |  |  |
|  |  |  |  |  |  |  |  |  |  |
| Abd-Alazeez(2013)[14] | Biopsy(WG) | T2+DWI+DCE | 108 | 26 | 43 | 8 | 31 | 0.76 | 0.42 |
| Aydin(2012)[15] | Biopsy(WG) | DCE | 216 | 54 | 30 | 72 | 60 | 0.43 | 0.67 |
|  |  | DWI | 210 | 34 | 17 | 82 | 77 | 0.29 | 0.82 |
|  |  | MRS | 246 | 98 | 53 | 44 | 51 | 0.69 | 0.49 |
|  |  | T2 | 252 | 67 | 34 | 80 | 71 | 0.46 | 0.68 |
| Baur(2014)[16] | Biopsy(WG) | DCE | 113 | 19 | 14 | 11 | 69 | 0.63 | 0.83 |
|  |  | DW | 113 | 26 | 6 | 4 | 77 | 0.87 | 0.93 |
|  |  | T2 | 113 | 21 | 6 | 9 | 77 | 0.70 | 0.93 |
|  |  | T2+DWI+DCE | 113 | 19 | 7 | 11 | 76 | 0.63 | 0.92 |
| Chabanova(2011)[17] | Biopsy(WG) | DCE | 258 | 73 | 34 | 76 | 75 | 0.49 | 0.69 |
|  |  | T2 | 258 | 107 | 49 | 42 | 60 | 0.72 | 0.55 |
| Iwazawa(2011)[18] | Biopsy(WG) | DCE | 1424 | 168 | 188 | 150 | 918 | 0.53 | 0.83 |
|  |  | DCE+DWI | 1424 | 232 | 219 | 86 | 887 | 0.73 | 0.80 |
|  |  | DWI | 1434 | 238 | 233 | 80 | 883 | 0.75 | 0.79 |
|  | Biopsy(PZ) | DCE | 712 | 118 | 106 | 66 | 422 | 0.64 | 0.80 |
|  |  | DCE+DWI | 709 | 149 | 117 | 32 | 411 | 0.82 | 0.78 |
|  |  | DWI | 712 | 150 | 114 | 34 | 414 | 0.82 | 0.78 |
| Kim CK(2006)[19] | Biopsy(PZ) | DCE | 120 | 47 | 7 | 17 | 49 | 0.73 | 0.88 |
|  |  | T2 | 120 | 35 | 7 | 29 | 49 | 0.55 | 0.88 |
| Kim JK(2005)[20] | Biopsy(WG) | DCE | 954 | 415 | 93 | 19 | 427 | 0.96 | 0.82 |
|  |  | T2 | 954 | 283 | 210 | 151 | 310 | 0.65 | 0.60 |
|  | Biopsy(PZ) | DCE | 636 | 276 | 9 | 13 | 338 | 0.96 | 0.97 |
|  |  | T2 | 636 | 218 | 163 | 71 | 184 | 0.75 | 0.53 |
|  | Biopsy(TZ) | DCE | 318 | 139 | 84 | 6 | 89 | 0.96 | 0.51 |
|  |  | T2 | 318 | 65 | 47 | 80 | 126 | 0.45 | 0.73 |
| Kitajima(2010)[21] | Biopsy(WG) | T2 | 424 | 60 | 28 | 39 | 297 | 0.61 | 0.91 |
|  |  | T2+DCE | 424 | 76 | 22 | 23 | 303 | 0.77 | 0.93 |
|  |  | T2+DWI | 424 | 75 | 19 | 24 | 306 | 0.76 | 0.94 |
|  |  | T2+DWI+DCE | 424 | 80 | 14 | 19 | 311 | 0.81 | 0.96 |
| Kozlowski(2010)[22] | Biopsy(WG) | DCE | 204 | 17 | 17 | 10 | 160 | 0.63 | 0.90 |
| Reisaeter (2015)[23]^1^ | Biopsy(WG) | DCE | 1695 | 165 | 152 | 198 | 1180 | 0.45 | 0.89 |
|  |  | DW | 1696 | 183 | 174 | 181 | 1158 | 0.50 | 0.87 |
|  |  | T2 | 1695 | 161 | 201 | 203 | 1130 | 0.44 | 0.85 |
|  |  | T2+DWI+DCE | 1699 | 196 | 191 | 169 | 1143 | 0.54 | 0.86 |
|  | Biopsy(PZ) | DCE | 1132 | 158 | 137 | 152 | 685 | 0.51 | 0.83 |
|  |  | DW | 1133 | 174 | 143 | 136 | 680 | 0.56 | 0.83 |
|  |  | T2 | 1133 | 154 | 172 | 157 | 650 | 0.50 | 0.79 |
|  |  | T2+DWI+DCE | 1134 | 188 | 166 | 123 | 657 | 0.60 | 0.80 |
|  | Biopsy(TZ) | DCE | 563 | 7 | 15 | 46 | 495 | 0.13 | 0.97 |
|  |  | DW | 563 | 9 | 31 | 45 | 478 | 0.17 | 0.94 |
|  |  | T2 | 562 | 7 | 29 | 46 | 480 | 0.13 | 0.94 |
|  |  | T2+DWI+DCE | 565 | 8 | 25 | 46 | 486 | 0.15 | 0.95 |
| Portalez(2010)[24] | Biopsy(PZ) | DCE | 408 | 12 | 24 | 29 | 343 | 0.29 | 0.93 |
|  |  | DWI | 408 | 16 | 15 | 25 | 352 | 0.39 | 0.96 |
|  |  | T2 | 408 | 20 | 48 | 21 | 319 | 0.49 | 0.87 |
| Puech(2009)[25] | Biopsy(WG) | T2+DCE | 664 | 68 | 22 | 144 | 430 | 0.32 | 0.95 |
| Rosenkrantz(2012)[26] | Biopsy(WG) | T2+DWI+DCE | 84 | 15 | 10 | 8 | 51 | 0.65 | 0.84 |
|  | patient | T2+DWI+DCE | 42 | 15 | 7 | 0 | 20 | 1.00 | 0.74 |
| Rosenkrantz(2015)[27]^2^ | Biopsy(TZ) | T2 | 636 | 21 | 15 | 41 | 559 | 0.34 | 0.97 |
|  |  | T2+DWI | 636 | 46 | 10 | 16 | 564 | 0.74 | 0.98 |
|  |  | T2+DWI+DCE | 636 | 46 | 14 | 16 | 560 | 0.74 | 0.98 |
| Tamada (2008)[28] | Biopsy(WG) | DCE | 320 | 59 | 14 | 70 | 177 | 0.46 | 0.93 |
|  |  | DWI | 320 | 73 | 19 | 56 | 172 | 0.57 | 0.90 |
|  |  | T2 | 338 | 66 | 18 | 63 | 191 | 0.51 | 0.91 |
|  |  | T2+DWI+DCE | 320 | 89 | 29 | 40 | 162 | 0.69 | 0.85 |
| Tamada (2011)[29] | Biopsy(WG) | DCE | 400 | 44 | 15 | 59 | 282 | 0.43 | 0.95 |
|  |  | DWI | 400 | 39 | 11 | 64 | 286 | 0.38 | 0.96 |
|  |  | T2 | 400 | 37 | 8 | 66 | 289 | 0.36 | 0.97 |
|  |  | T2+DWI+DCE | 400 | 55 | 20 | 48 | 277 | 0.53 | 0.93 |
|  | patient | DCE | 50 | 26 | 3 | 9 | 12 | 0.74 | 0.80 |
|  |  | DWI | 50 | 24 | 2 | 11 | 13 | 0.69 | 0.87 |
|  |  | T2 | 50 | 21 | 2 | 14 | 13 | 0.60 | 0.87 |
|  |  | T2+DWI+DCE | 50 | 29 | 3 | 6 | 12 | 0.83 | 0.80 |
| Turkbey(2011)[30] | Biopsy(WG) | DCE | 605 | 23 | 24 | 33 | 525 | 0.41 | 0.96 |
|  |  | DWI | 605 | 33 | 112 | 23 | 437 | 0.59 | 0.80 |
|  |  | MRS | 608 | 17 | 9 | 41 | 541 | 0.29 | 0.98 |
|  |  | T2 | 605 | 32 | 119 | 24 | 430 | 0.57 | 0.78 |
|  |  | T2+DW+DCE+MRS | 605 | 34 | 150 | 22 | 399 | 0.61 | 0.73 |
| Van den Bergh(2013)[31] | Biopsy(WG) | DCE | 1754 | 176 | 69 | 532 | 977 | 0.25 | 0.93 |
|  |  | DWI | 1752 | 312 | 81 | 396 | 963 | 0.44 | 0.92 |
|  |  | T2 | 1752 | 221 | 55 | 487 | 989 | 0.31 | 0.95 |
|  |  | T2+DWI+DCE | 1752 | 405 | 155 | 303 | 889 | 0.57 | 0.85 |
| Weidner (2011)[32]^3^ | Biopsy(PZ) | DCE | 32 | 11 | 8 | 3 | 10 | 0.79 | 0.56 |
|  |  | DWI | 32 | 11 | 11 | 3 | 7 | 0.79 | 0.39 |
|  |  | MRS | 31 | 11 | 4 | 3 | 13 | 0.79 | 0.76 |
|  |  | T2 | 32 | 10 | 10 | 4 | 8 | 0.71 | 0.44 |
| Yoshizako(2008)[33] | Biopsy(TZ) | T2 | 42 | 16 | 5 | 10 | 11 | 0.62 | 0.69 |
|  |  | T2+DCE | 42 | 18 | 5 | 8 | 11 | 0.69 | 0.69 |
|  |  | T2+DWI | 42 | 21 | 2 | 5 | 14 | 0.81 | 0.88 |
|  |  | T2+DWI+DCE | 45 | 18 | 1 | 8 | 18 | 0.69 | 0.95 |
| Yu(2008)[34]^4^ | Biopsy(WG) | DCE | 126 | 28 | 4 | 32 | 62 | 0.47 | 0.94 |
|  |  | T2 | 126 | 22 | 10 | 38 | 56 | 0.37 | 0.85 |
| Zhang(2014)[35] | patient | DCE | 72 | 40 | 7 | 4 | 21 | 0.91 | 0.75 |
|  |  | T2 | 75 | 28 | 13 | 16 | 18 | 0.64 | 0.58 |
| Ferda(2013)[36] | patient | T2+DWI+DCE | 164 | 82 | 28 | 2 | 52 | 0.98 | 0.65 |
| Vilanova(2011)[37] | patient | DWI | 70 | 31 | 7 | 7 | 25 | 0.82 | 0.78 |
|  |  | T2+DWI | 70 | 31 | 7 | 7 | 25 | 0.82 | 0.78 |
|  |  | T2+DWI+DCE+MRS | 70 | 36 | 6 | 2 | 26 | 0.95 | 0.81 |
|  |  | T2+MRS | 70 | 30 | 6 | 8 | 26 | 0.79 | 0.81 |
| Watanabe(2010)[38] | patient | T2+DCE | 43 | 23 | 2 | 3 | 15 | 0.88 | 0.88 |
| Haffner(2011)[39] | patient | T2+DCE | 555 | 252 | 99 | 50 | 154 | 0.83 | 0.61 |

1. The data extracted from observer 2 result

2. The data extracted from read 1 result

3. The data extracted from consensus reading result

4. The data extracted from overall result

Abbreviations:

DCE: dynamic contrast-enhanced magnetic resonance. DWI: diffusion weighted imaging. T2: T2 weighted imaging. MRS: magnetic resonance spectroscopy. WG: whole gland. PZ: peripheral zone. TZ: transition zone.
