# Supplementary material for: Accuracy of dynamic contrast-enhanced magnetic resonance imaging in the diagnosis of prostate cancer: systematic review and meta-analysis
Source: Oncotarget. 2017 Aug 17;8(44):77975–89. doi: 10.18632/oncotarget.20316 (PMC5652829; doi:10.18632/oncotarget.20316)
Supplement: Supplementary file 3 [file oncotarget-08-77975-s003.docx]

Supplementary Table 5: Search strategies

EMBASE search strategy - January 2000 to [September](https://cn.bing.com/dict/clientsearch?mkt=zh-CN&setLang=zh&form=BDVEHC&ClientVer=BDDTV3.5.1.4320&q=%E4%B9%9D%E6%9C%88) 2016

#1 ‘prostatic’

#2 ‘prostate tumor’

#3 ‘prostate cancer’

#4 ‘prostate carcinoma’

#5 #2 OR #3 OR #4 OR #5

#6 magnetic AND resonance AND imaging

#7 ‘magnetic resonance imaging’

#8 ‘magnetic resonance methods’

#9 ‘magnetic resonance’

#10 ‘MRI’

#11 #6 OR #7 OR #8 OR #9 OR #10

#12 ‘contrast medium’

#13 ‘contrast’

#14 ‘DCE’

#15 ‘DCE MRI’

#16 ‘dynamic’

#17 ‘contrast enhanced tomography’

#18 ‘contrast enhanced'

#19 ‘enhanced’

#20 #12 OR #13 OR #14 OR #15 OR #16

#21 #5 AND #11 AND #20

#22 #21 AND [humans]/lim AND [english]/lim

PubMed search strategy - January 2000 to [September](https://cn.bing.com/dict/clientsearch?mkt=zh-CN&setLang=zh&form=BDVEHC&ClientVer=BDDTV3.5.1.4320&q=%E4%B9%9D%E6%9C%88) 2016

#1 Prostatic [MeSH Major Topic]

#2 Prostatic Neoplasms [MeSH Major Topic]

#3 prostate cancer

#4 prostate carcinoma

#5 #1 OR #2 OR #3 OR #4

#6 Magnetic Resonance Imaging [MeSH Major Topic]

#7 Magnetic Resonance methods [MeSH Major Topic]

#8 Magnetic Resonance Imaging [Text word]

#9 Magnetic Resonance

#10 Magnetic

#11 MRI

#12 #6 OR #7 OR #8 OR #9 OR #10 OR #11

#13 Contrast Media [MeSH Major Topic]

#14 Contrast [MeSH Major Topic]

#15 Contrast

#16 DCE

#17 DCE-MRI

#18 Dynamic

#19 contrast-enhanced

#20 enhanced

#21 #13 OR #14 OR #15 OR #16 OR #17 OR #18 OR #19 OR #20

#22 ENGLISH [Language]

#23 #5 OR #12 OR #21 OR #22

Cochrane library and CENTRAL search strategy - January 2000 to [September](https://cn.bing.com/dict/clientsearch?mkt=zh-CN&setLang=zh&form=BDVEHC&ClientVer=BDDTV3.5.1.4320&q=%E4%B9%9D%E6%9C%88) 2016

#1 prostatic

#2 prostate tumor

#3 prostate cancer

#4 prostate carcinoma

#5 #1 OR #2 OR #3 OR #4

#6 magnetic resonance imaging

#7 magnetic resonance methods

#8 magnetic resonance

#9 MRI

#10 #6 OR #7 OR #8 OR #9

#11 contrast medium

#12 contrast

#13 DCE

#14 DCE MRI

#15 enhanced

#16 #11 OR #12 OR #13 OR #14 OR #15

#17 #5 AND #10 AND #16
