# Supplementary material for: Accuracy of dynamic contrast-enhanced magnetic resonance imaging in the diagnosis of prostate cancer: systematic review and meta-analysis
Source: Oncotarget. 2017 Aug 17;8(44):77975–89. doi: 10.18632/oncotarget.20316 (PMC5652829; doi:10.18632/oncotarget.20316)
Supplement: Supplementary file 4 [file oncotarget-08-77975-s004.docx]

Supplementary Table 6: Quality assessment of diagnostic accuracy studies (QUADAS-2) checklist

| Domain 1: patient selection | |  | | |
| --- | --- | --- | --- | --- |
| A. Risk of Bias | | Yes | No | Unclear |
| Patient Sampling | |  | | |
| 1. Was a consecutive or random sample of patients enrolled? | |  |  |  |
| 2. Was a case-control design avoided? | |  |  |  |
| 3. Did the study avoid inappropriate exclusions? | |  |  |  |
|  | | RISK |  | |
|  |  | Low | High | Unclear |
| Could the selection of patients have introduced bias? | |  | | |
| B. Concerns regarding applicability | |  |  |  |
| Patient characteristics and setting |  | Concern |  | |
|  |  | Low | High | Unclear |
| Is there concerns that the included patients and setting do not match the review question? | |  | | |
| Domain 2: index & comparator test(s) | |  |  |  |
| A. Risk of Bias | |  |  |  |
|  | | Yes | No | Unclear |
| 4. Were the index test results interpreted without knowledge of the results of the reference standard? | |  | | |
| 5. If a threshold was used, was it pre-specified? | |  |  |  |
| 6. For a test requiring subjective interpretation, was it interpreted by someone experienced in interpreting such tests? | |  |  |  |
|  | | RISK |  | |
|  |  | Low | High | Unclear |
| Could the reference standard, its conduct, or its interpretation have introduced bias? | |  | | |
| B. Concerns regarding applicability | |  |  |  |
|  | | Concern | `` |  |
|  |  | Low | High | Unclear |
| Are there concerns that the index test, its conduct, or interpretation differ from the review question? | |  | | |
| Domain 3:Reference Standard | |  |  |  |
| A. Risk of Bias | |  |  |  |
| Target condition and reference standard(s) |  | Yes | No | Unclear |
| 7. Is the reference standards likely to correctly classify the target condition? |  |  | | |
| 8. Were the reference standard results interpreted without knowledge of the results of the index test? | |  |  |  |
| 9. Were the results of the reference standard test interpreted by someone experienced in interpreting such tests? | |  |  |  |
| B. Concerns regarding applicability |  |  |  |  |
|  | | Concern |  | |
|  |  | Low | High | Unclear |
| Are there concerns that the target condition as defined by the reference standard does not match the question? | |  | | |
| Domain 4:Flow and timing | |  |  |  |
| A. Risk of Bias | |  |  |  |
|  | | RISK |  | |
|  |  | Low | High | Unclear |
| Was there an appropriate interval between index test and reference standard? | |  | | |
| 10. Did all patients receive the same reference standard? | |  |  |  |
| 11. Were all patients included in the analysis? | |  |  |  |
| 12. Could the patient flow have introduced bias? | |  |  |  |
|  | | Concern |  | |
|  |  | Low | High | Unclear |
| Could the patient flow have introduced bias? | |  | | |
